# Aortic Response to Strength Training and *Spirulina platensis* Dependent on Nitric Oxide and Antioxidants

**DOI:** 10.3389/fphys.2018.01522

**Published:** 2018-10-31

**Authors:** Aline de Freitas Brito, Alexandre S. Silva, Alesandra A. de Souza, Paula B. Ferreira, Iara L. L. de Souza, Layanne C. da Cunha Araujo, Gustavo da Silva Félix, Renata de Souza Sampaio, Maria da Conceição C. Silva, Renata L. Tavares, Reabias de Andrade Pereira, Manoel Miranda Neto, Bagnólia A. da Silva

**Affiliations:** ^1^School of Physical Education, University of Pernambuco, Recife, Brazil; ^2^Post-Graduation Program in Physical Education UPE/UFPB, Recife, Brazil; ^3^Physical Education Department, Health Sciences Center, Federal University of Paraiba, João Pessoa, Brazil; ^4^Federal University of Tocantins, Licentiate in Physical Education, Tocantinópolis, Brazil; ^5^Postgraduate Program in Natural and Synthetic Products Bioactive, Health Sciences Center, Federal University of Paraiba, João Pessoa, Brazil; ^6^Department of Biophysics and Physiology, Institute of Biomedical Sciences, University of São Paulo, São Paulo, Brazil; ^7^Laboratory of Studies of Physical Training Applied to the Performance and the Health, Health Sciences Center, Federal University of Paraiba, João Pessoa, Brazil; ^8^Pharmaceutical Sciences Department, Health Sciences Center, Federal University of Paraiba, João Pessoa, Brazil

**Keywords:** *Spirulina platensis*, exercise, vasorelaxation, vasoconstriction, antioxidant activity

## Abstract

Studies have shown that supplementation with *Spirulina platensis* improves vascular reactivity. However, it is unclear whether in association with strength training this effect can be enhanced. Thus, this study aimed to determine the effects of strength training and *S. platensis* on the reactivity of the aorta from Wistar rat and the possible mechanisms involved. The animals were supplemented with *S. platensis* and divided into sedentary (SG, SG50, SG150, and SG500) and trained groups (TG, TG50, TG150, and TG500). Nitrite, malondialdehyde (MDA) and antioxidant activity were determined by biochemical assays. To evaluate vascular response, cumulative concentration—response curves to phenylephrine (PHE) and acetylcholine (ACh) were constructed. L-NAME was used to assess the participation of nitric oxide (NO). It was observed that the PHE contractile potency was reduced in TG50, TG150, and TG500 groups compared to SG50, SG150, and SG500 groups, respectively. However, the presence of L-NAME increased the contractile response in all groups. Strength training potentiated the increase in relaxing activity induced by *S. platensis*, where the pCE_50_ values of ACh increased in TG150 and TG500. These responses were accompanied by increased nitrite production, MDA reduction and increased antioxidant activity in the aorta of both TG150 and TG500 groups. Thus, the present study demonstrated that combined with strength training, *S. platensis* potentiates vascular improvement through the participation of NO and reduction of oxidative stress.

## Introduction

Increase in vasoconstrictor response, decrease in vasodilatory capacity and increase in the production of reactive oxygen species (ROS) as a result of decreased antioxidant capacity are associated with cardiovascular risk factors, such as hypertension (Virdis et al., 2013).

Moreover, research has shown that physical training performed regularly promotes improvement of endothelial function by increasing relaxant response and decreasing contractile reactivity (Silva et al., 2011; Blanco-Rivero et al., 2013; Trinity et al., 2013), as well as by having a protective effect against oxidative stress, reducing the production of free radicals and enhancing the activity of antioxidant enzymes (Ferrara et al., 2008; Roque et al., 2013). The performance of aerobic (triathlon), mixed aerobic-anaerobic (soccer), and anaerobic (sprint running) exercises was associated to increase in antioxidant activity in human endothelial cells (Conti et al., 2012).

In relation to strength training, some human research that used indirect methods have identified similar responses in the vascular improvement (Trinity et al., 2013; Beck et al., 2014; Brito et al., 2014; Choi et al., 2016) and antioxidant action (Scheffer et al., 2012). However, studies that explain mechanisms underlying these responses through direct methods, such as vascular reactivity in an animal model are scarce. There are only three studies described by Figard et al. (2006), Harris et al. (2010), and Araujo et al. (2013), however, there was no consensus between protocols. In this view, little attention has been given to the contractile reactivity and possible mechanisms of action.

In addition to the positive action of physical training on the oxidative stress process, bioactivity compounds consumed in the diet or used as supplements provide protection against negative consequences to health (Lau et al., 2007; Scapagnini et al., 2011; Davinelli et al., 2016). In the literature, different studies have consolidated the nature benefit of compounds which are major sources of antioxidants as resveratrol (Barrajon-Catalan et al., 2014; Davinelli et al., 2017), Cocoa polyphenols and gamma-3 fatty acids (Manach et al., 2004; Andres-Lacueva et al., 2008; Davinelli et al., 2018). Therefore, we can highlight *S. platensis*, blue-green algae, due to its diverse biological activities. Indeed, pre-clinical and clinical studies have shown that the intake of *S. platensis* can exert antioxidant action (Wu et al., 2012) and reduction of endothelial dysfunction (Huang et al., 2005). For these reasons, the first hypothesis of this study was that the improvement of antioxidant capacity and endothelial function could result in better response to relaxing agents and decreased vascular reactivity to contractile agents. Besides that, the second hypothesis was that the best antioxidant and endothelial action combined with resistance training could enhance the effects on vascular reactivity in response to exercise.

Nevertheless, the potential synergistic effects of strength training and *S. platensis* have not yet been described in the literature as vascular protector and antioxidant. Therefore, the aim of this study was to investigate the effects of strength training and *S. platensis* supplementation on vascular reactivity in the isolated aorta of Wistar rats and to observe if nitric oxide and oxidative stress were involved in this response.

## Materials and methods

### Animals

Male Wistar rats (*Rattus norvegicus*), weighing between 250 and 300 g, were obtained from the Bioterium Prof. Thomas George of the Research Institute for Drugs and Medicines of UFPB. Before the experiments, the animals were kept under a balanced diet for feed base (Labina®) with free access to water, ventilation and controlled and constant temperature (21 ± 1°C), daily exposed to a 12 h light-dark cycle, and the light period from 6 a.m. to 18 p.m. All experiments were performed in a period of 8 a.m. to 20 p.m., following the guidelines for the ethical use of animals (Sherwin et al., 2005). All experiments were previously approved by the Ethics Committee on Animal Use from the Center of Biotechnology (CEUA/CBiotec) with certificate number 0511/13.

The animals were divided into sedentary groups and groups subjected to the strength training protocol supplemented with *S. platensis* (50, 150, and 500 mg/kg) or saline solution orally. Thus, the work had eight groups with 20 rats each: saline sedentary group (SG, control), sedentary group 50 (SG50), sedentary group 150 (SG150), sedentary group 500 (SG500), saline trained group (TG), trained group 50 (TG50), trained group 150 (TG150), and trained group 500 (TG500).

### Substances

Calcium chloride dihydrate (CaCl_2_.2H_2_O), potassium chloride (KCl) and sodium bicarbonate (NaHCO_3_) were purchased from Vetec (Rio de Janeiro - RJ, Brazil). Glucose (C_6_H_12_O_6_), magnesium sulfate heptahydrate, (MgSO_4_.7H_2_O), hydrochloric acid (HCl) and monobasic potassium phosphate (KH_2_PO_4_) were obtained from Nuclear (Porto Alegre - RS, Brazil). Sodium chloride (NaCl) was purchased from Dynamics (Diadema-SP, Brazil). The acetylcholine (ACh) was obtained from Merck (Brazil). Phenylephrine (PHE) was acquired from Pfizer (USA). *N*ω-nitro-L-arginine methyl ester (L-NAME) was purchased from Sigma-Aldrich (Brazil). The ethylenediaminetetraacetic acid (EDTA) (1:250) was purchased from BioTécnica-Advanced Biotechnology (Brazil). The carbogen mixture (95% O_2_ and 5% CO_2_) was obtained from White Martins (Brazil). All substances were weighed on an analytical balance, model AG 200 GEHAKA (São Paulo-SP, Brazil).

### Preparation and administration of *Spirulina platensis*

*S. platensis* in powder form was obtained from Bio-Engineering Dongtai Top Co. LTD. Laboratory (Nanjing, China) (lot n°. 20130320). A sample was analyzed by Pharma Nostra Quality Control Laboratory (Anapolis - GO, Brazil) (lot n°. 1308771A) to certify that the extract obtained was from *S. platensis*. The Dilecta Manipulation Drugstore (João Pessoa) was responsible for the handling and preparation of the dried powder (lot n°. 20121025).

The *S. platensis* powder was dissolved in saline solution (NaCl 0.9%) each day for the doses preparation of 50, 150, and 500 mg/kg and administered in the animals. The supplementations were carried out for a period of 8 weeks for all doses (50, 150, and 500 mg/kg/day) (adapted from Juárez-Oropeza et al., 2009). The administration was made orally in the period of 12 p.m. to 14 p.m., using stainless steel needles for gavage (BD-12, Insight, Ribeirão Preto, SP). For groups submitted to strength training, the supplementation was performed 30 min prior to the exercise session (Stefani et al., 2014).

### Strength training program

The animals of the strength training groups were subjected to a specific jumping program in a PVC cylinder, 30 cm in diameter and 70 cm high, containing water. The water depth in the tanks was 50 cm, approximately twice the length of the rats, aimed at limiting their attempt to climb the wall to cling to the cylinder edge. Before starting the exercise, the water was heated to about 32°C, a comfortable temperature for rats exercise (Marqueti et al., 2006).

Strength training was based on the jumping protocol in water, developed by Marqueti et al. (2006). Briefly, the protocol consists of 4 series of 10 to 12 repetitions, with 30-s intervals between sets, with a progressively increasing load adjusted according to body weight. The load was applied to the chest of the animals using a special vest that allowed the jumps without loosening of the load from the body or preventing their movement. All exercise sessions were always held from 12 p.m. to 14 p.m.

Strength training and adjusting the overload was carried out as follows:

- Week of adaptation—three days of alternate exercise sessions with a load corresponding to 50% of animal body weight, so that the number of sets and repetitions is adjusted every session and a 30-s rest between sets is added (1st day: 2 sets x 5 jumps; 2nd day: 4 sets x 5 jumps; and 3rd day: 4 sets x 9 jumps).
- 1st and 2nd weeks—4 sets of 10 jumps, with a 30-s rest between the series and an overload of 50% of the animal body weight.
- 3rd and 4th weeks—4 sets of 10 jumps, with a 30-s rest between the series and an overload of 60% of body weight.
- 5th and 6th weeks—the 4 sets of 10 jumps remained, with a 30 s rest between the series and an overload of 80% of the animal body weight.
- 7th and 8th weeks—the 4 sets of 12 jumps remained, with a 30 s rest between the series and an overload of 80% of body weight.

During each exercise session, it was determined the series runtime that the animal spent to perform it with the goal of evaluating the exercise effectiveness on muscle strength.

The animals were euthanized by guillotine 48 h after the last training session and supplementation, to eliminate the acute exercise effect on reactivity.

Among the sedentary groups, only the supplemented with the 500 mg/kg dose had a significant reduction in body mass as of the 6th week of intervention. In 6th and 7th weeks, their reduction was significant when compared to SG and in 8th week when compared to SG, SG50, and SG150. Among the trained groups, the groups supplemented with S. platensis at doses of 150 mg/kg and 500 mg/kg significantly reduced body mass after 6 weeks. However, TG150 showed only significant reduction when compared to SG150 at the end of the 6th, 7th, and 8th weeks. While for TG500 the reductions were verified when compared with TG, TG50, TG150, and SG500 at the end of the 6th, 7th, and 8th weeks of intervention.

### Muscular performance

The strength training followed the progressivity principle of sports training for obtaining improved performance. Thus, this protocol can lead to trauma risk in muscle tissue resulting in local muscle inflammation, increase oxidative stress and muscle wasting (Schoenfeld, 2010).

Initially, to confirm the effectiveness of the exercise protocol used in this study, a muscle performance evaluation was made by the time that the animal expended to perform the exercise series during the intervention period. Furthermore, after 8 weeks training, lactate dehydrogenase (LDH) and creatine kinase (CK) were measured, in order to observe an increase in the activity of these enzymes, fact related to exercise intensity. Without these enzymatic increases, an exaggerated muscular stress and chronic negative effects can be observed.

### Determination of lactate dehydrogenase activity and creatine kinase activity

After euthanasia, 3 mL of blood was collected by cardiac puncture (Okafor et al., 2011) and, 1 mL of the collected blood was placed in test tubes without anticoagulant to obtain a serum for determination of CK activity and LDH (Siekmann et al., 2002). LDH and CK activities in serum using the commercial kit Labtest (Minas Gerais, Brazil). For CK and LDH assays, 20 μL of serum was added to 1 mL of the reagent according to kit instructions. Absorbance was read in an automatic analyzer, LabMax 240 premium, at 340 nm for CK and LDH, at room temperature.

### Concentration-response curves

After euthanasia, the thoracic aorta was carefully removed and cleaned to remove the connective and adipose tissues. The aorta was divided into segments of 3-5 mm in length, individually suspended by means of stainless steel clips in organ baths (6 mL) (Model OX 04) containing Krebs's solution (mM: NaCl 118.0, KCl 4.6, 1.1 KH_2_PO_4_, MgSO_4_ 5.7, CaCl_2_ 2.5, NaHCO_3_ 25.0, and glucose 11.0), pH 7.4 adjusted with 1 N HCl or NaOH solution (digital pH meter PG2000 GEHAKA-São Paulo, Brazil) at 37°C controlled by a thermostatic pump (BT-60 model, AVS Project-São Paulo, Brazil) and gassed with carbogen. The isometric tension was evaluated by isometric force transducers (TIM Model 05), coupled to an amplifier (AECAD04F model), connected to a digital acquisition system with AQCAD version 2.1.6 software to obtain the data, and the ANCAD software was used for analysis.

The preparations were stabilized for a period of 1 h and maintained under a resting tension of 1 g. During this period, the nutrient solution was renewed every 15 min to prevent interference of metabolites (Altura and Altura, 1970). After the stabilization period, a contraction was induced by 3 × 10^−7^ M PHE, and during the tonic component, 10^−6^ M ACh was added to determine endothelium integrity (Furchgott and Zawadki, 1980). The vascular endothelium was considered intact when aortic rings showed relaxation equal to or higher than 50% of total contraction (100%), and when relaxation was equal to or less than 10% of total contraction (100%), they were regarded as lacking functional endothelium.

After endothelium integrity verification, to assess the contractile response of rat aorta with or without functional endothelium, the preparations were washed, and after 30 min, a cumulative concentration-response curve was obtained with PHE (10^−9^–10^−3^ M) in the rat aortic rings of all groups (Juárez-Oropeza et al., 2009).

After endothelium integrity verification, the relaxing response of rat aorta with functional endothelium was evaluated. The preparations were washed and after 30 min a new contraction with 3 × 10^−7^ M PHE was induced, and ACh (10^−11^–10^−4^ M) was added cumulatively to the organ bath during the tonic component of contraction (Heylen et al., 2008).

#### Nitric oxide signaling pathway evaluation

After assessing endothelium integrity, the preparations were washed, and 30 min later, the tissue was incubated with L-NAME (10^−4^ M), a non-selective competitive inhibitor of nitric oxide synthase (NOS) (Juárez-Oropeza et al., 2009) for 30 min, and a cumulative concentration-response curve to PHE (10^−11^–10^−3^ M) was obtained in aortic preparations of all groups. The results were evaluated by comparing the amplitude of the contractile response of rat aorta with the functional endothelium of groups treated with *S. platensis* with that obtained by the average of the maximum amplitudes of control curves in the presence and absence of L-NAME.

### Biochemical measurements

After euthanasia, 3 mL of blood was collected by cardiac puncture (Okafor et al., 2011) and, 2 mL were placed in test tubes with anticoagulant (EDTA) to obtain plasma in order to determine nitrite, malondialdehyde (MDA) and antioxidant activity, using commercial kit. After collection, the samples were centrifuged at 1,207 g for 15 min in a CENTRIBIO centrifuge; model 80-2B-15ML (Guarulhos- SP, Brazil). The supernatant was transferred to Eppendorf tubes and refrigerated at −20°C until analysis. All analyzes were performed within 7 days after blood collection.

Aortic fragments of 8 mm in length were obtained for measuring nitrite, MDA, and antioxidant activity, and heart samples for MDA and antioxidant activity measurements. These tissues were quickly removed, cleaned with Krebs's solution to remove the remaining blood, placed in Eppendorf tubes and stored in a freezer at −80°C until analysis.

#### Evaluation of nitrite in plasma and aorta

The concentration of nitrite was determined by the Griess method proposed by Green et al. (1981). The Griess reagent was prepared using equal parts of 5% phosphoric acid, 0.1% N-1-naphylenediamine (NEED) and 1% sulfanilamide in 5% phosphoric acid and distilled water. The assay was performed by mixing 500 μL of plasma or aorta homogenate with 500 μL of Griess reagent and reading the absorbance at 560 nm after 10 min. The blank was prepared by mixing 100 μL of reagent and 100 μL of 10% potassium phosphate buffer, and the standard curve was constructed using serial dilutions of sodium nitrite (NaNO_2_) (100, 50, 25, 12.5, 6.25, 3.12; 1.56 mM). Absorbance was read in an SP-220 UV-Vis spectrophotometer (Biospectro, Curitiba-PR, Brazil).

#### Malondialdehyde determination

For the analysis of MDA in aorta and heart, tissues were homogenized with 10% KCl in proportions of 1:1. Plasma and homogenate (250 μL) were incubated in a water bath at 37°C for 60 min. The samples were precipitated with 400 μL of 35% perchloric acid and centrifuged at 26,295 g for 10 min at 4°C. The supernatant was transferred to new Eppendorf tubes, and 400 μL of 0.6% thiobarbituric acid was added. The tubes were incubated at 95–100°C for 30 min. After cooling, the material was read in a spectrophotometer at 532 nm (Ohkawa et al., 1979).

MDA concentration in each plasma sample or tissue was determined using a standard curve of MDA, obtained on the basis of a standard solution (1 μL of 1,1,3,3-tetramethoxypropane in 70 mL of distilled water) diluted in series (250, 500, 750, 1000, 1250, 1500, 1750, 2000, 2250, 2500, 2750, and 3000 μL of distilled water). In tissues, the absorbance values obtained were normalized by the given sample volume dry weight.

#### Antioxidant activity evaluation

The procedure was based on the method described by Brand-Williams et al. (1995) using an aliquot of 1.25 mg DPPH diluted in 100 mL of ethanol, kept under refrigeration and protected from light (aluminum foil or with amber glass). In centrifugation appropriate tubes, 3.9 mL of DPPH solution was added along with 100 μL of homogenate with KCl or plasma. The tubes were vortexed and left to stand for 30 min. Then, they were centrifuged at 13,416 g at 20°C for 15 min, and the supernatant absorbance was read in a spectrophotometer at 515 nm. The results were expressed as oxidation inhibition percentage according to the following formula: AOA = 100-([DPPH • R]_T_/[DPPH • R]_B_ 100), where [DPPH • R]_t_ and [DPPH • R]_B_ are the concentration of DPPH • remaining after 30 min, measured in the sample (t) and blank (B) prepared with distilled water.

### Statistical analysis

The results obtained were expressed as the mean and standard error of the mean (SEM). These results were analyzed statistically using Student's *t*-test or two-way analysis of variance (ANOVA) followed by Bonferroni's post-test, and the differences between means were considered significant when *p* < 0.05. The pCE_50_ values were calculated by non-linear regression (Neubig et al., 2003). The maximum effect (E_max_) values were obtained by averaging maximum percentages of contraction or relaxation. All results were analyzed by GraphPad Prism® version 5.01 (GraphPad Software Inc., San Diego, CA, USA).

## Results

### Effect of strength training and supplementation with *S. platensis* on muscle performance

**Figure 1:**
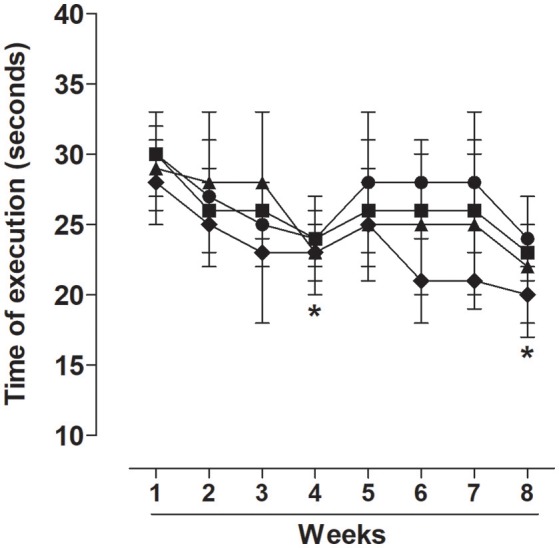
Time of execution of exercise in eight-week training sessions for TG (•), TG50 (■), TG150 (▴) and TG500 (♦) groups. The symbols and vertical bars represent the mean and SEM, respectively (n = 20). Two-way ANOVA followed by Bonferroni's post-test, ^*^p < 0.05 (TG 1st week vs. TG 4th or 8th week, TG50 1st week vs. TG50 4th or 8th week, TG150 1st week vs. TG150 4th or 8th week, TG500 1st week vs. TG500 4th or 8th week).

The groups submitted to the strength training program improved their exercise execution time in the 4th and 8th weeks of training in relation to the first week in TG (24.0 ± 2.0 and 24.0 ± 3.0 vs. 30.0 ± 3.0 s, respectively), TG50 (24.0 ± 3.0 and 23.0 ± 2.0 vs. 30.0 ± 2.0 s, respectively), TG150 (23.0 ± 3.0 and 22.0 ± 5.0 vs. 29.0 ± 3.0 s, respectively), and TG500 (23.0 ± 2.0 and 20.0 ± 2.0 vs. 28.0 ± 3.0 s, respectively) (Figure 1).

### Effect of strength training and supplementation with *S. platensis* on muscle stress and the cellular lysis

**Figure 2:**
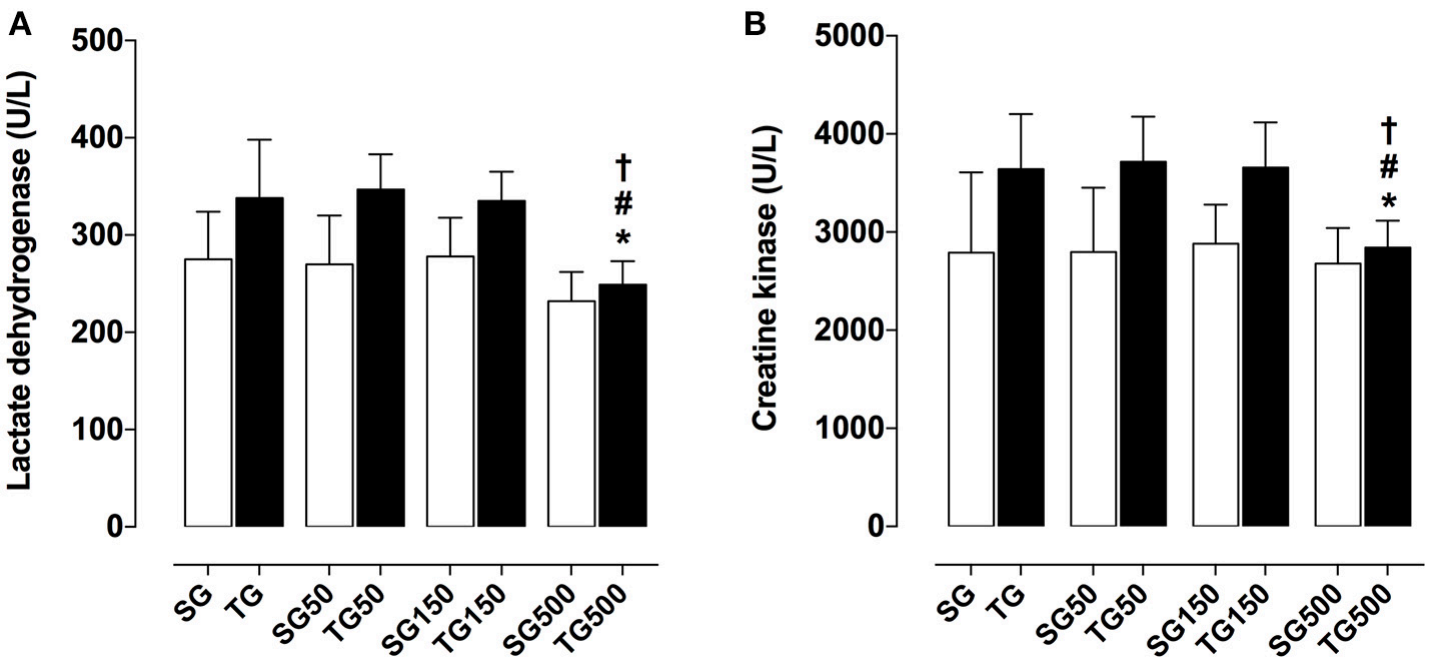
Concentration of lactate dehydrogenase **(A)** and creatine kinase **(B)** from SG, TG, SG50, TG50, SG150, TG150, SG500, and TG500 groups. The columns and vertical bars represent the mean and SEM, respectively (*n* = 8). Two-way ANOVA followed by Bonferroni's post-test, *p < 0.05 (TG vs. SG500), ^#^*p* < 0.05 (TG50 vs. TG500), ^†^*p* < 0.05 (TG150 vs. TG500).

Among the trained groups, only the supplemented with *S. platensis* at 500 mg/kg showed a reduction of LDH when compared to TG (250.0 ± 24.0 vs. 338.0 ± 60.0 U/L, respectively), TG50 (250.0 ± 24.0 vs. 347.0 ± 36.0 U/L, respectively) and TG150 (250.0 ± 24.0 vs. 335.0 ± 30.0 U/L, respectively) (Figure 2A). It was also observed that CK production was decreased in TG500, compared to TG (2842.0 ± 273.0 vs. 3643.0 ± 561.0 U/L, respectively), TG50 (2842.0 ± 273.0 vs. 3718.0 ± 460.0 U/L, respectively) and TG150 (2842.0 ± 273.0 vs. 3658.0 ± 460.0 U/L, respectively) (Figure 2B).

The muscle wasting markers LDH and CK in the trained groups did not change in relation to the untrained groups, indicating that the training load was well supported by the animals (Figure 2).

### Effect of strength training and supplementation with *S. platensis* on the contractile response induced by PHE in the presence of functional endothelium

**Figure 3:**
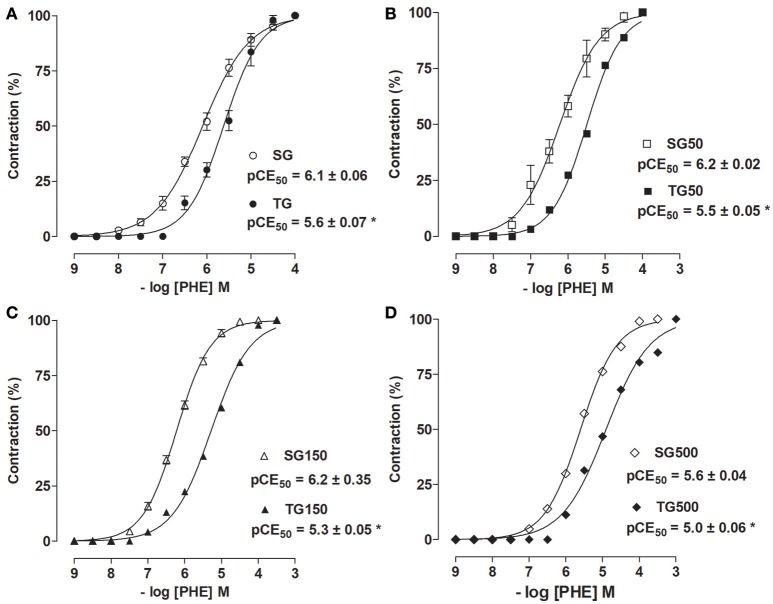
Contractile effect of PHE in SG (°), TG (•) (**A**), SG50 (□), TG50 (■) (**B**), SG150 (▵), TG150 (▴) **(C)**, SG500 (♢), and TG500 (♦) **(D)** groups in rat aorta in the presence of endothelium. The symbols and vertical bars represent the mean and SEM, respectively (*n* = 5). Student's *t*-test, *p < 0.05 (SG vs. TG, SG50 vs. TG50, SG150 vs. TG150, SG500 vs. TG500).

Among the sedentary groups supplemented with *S. platensis*, only the dose of 500 mg/kg shifted the cumulative concentration-response curve of PHE to right (pCE_50_ = 5.6 ± 0.04) compared to SG, SG50 and SG150 (pCE_50_ = 6.1 ± 0.06, 6.2 ± 0.02, and 6.2 ± 0.04, respectively). Among the animals subjected to strength training and supplementation with *S. platensis*, doses of 150 and 500 mg/kg shifted the concentration-response curve to the right compared to TG and TG50 (pCE_50_ = 5.3 ± 0.05 and 5.0 ± 0.06 vs. 5.6 ± 0.07, and 5.5 ± 0.05, respectively) (Figure 3).

It was observed that the contractile potency of PHE was reduced in TG (pCE_50_ = 5.6 ± 0.07) compared to SG (pCE_50_ = 6.1 ± 0.06) (Figure 3A). This reduction was also observed for TG50 vs. SG50 (pCE_50_ = 5.5 ± 0.05 vs. 6.2 ± 0.02, respectively, Figure 3B), TG150 vs. SG150 (pCE_50_ = 5.3 ± 0.05 vs. 6.2 ± 0.04, respectively, Figure 3C) and TG500 vs. SG500 (pCE_50_ = 5.0 ± 0.06 vs. 5.6 ± 0.04, respectively, Figure 3D).

The decrease in the contractile potency in SG150 was similar to that in TG (pCE_50_ = 6.2 ± 0.04 vs. 5.6 ± 0.07, respectively). In addition, the contractile potency induced by PHE was reduced in TG150 compared to TG and SG150 (pCE_50_ = 5.3 ± 0.05 vs. 6.2 ± 0.04, and 5.6 ± 0.07, respectively). In SG500, reduction in PHE contractile potency was similar to those in TG (pCE_50_ = 5.6 ± 0.07 vs. 5.6 ± 0.04, respectively). However, in TG500, the reduction was greater than in TG and SG (pCE_50_ = 5.0 ± 0.06 vs. 5.6 ± 0.07 vs. 5.6 ± 0.04, respectively) (Figure 3).

### Effect of strength training and supplementation with *S. platensis* on contractile response induced by PHE in absence of functional endothelium

**Figure 4:**
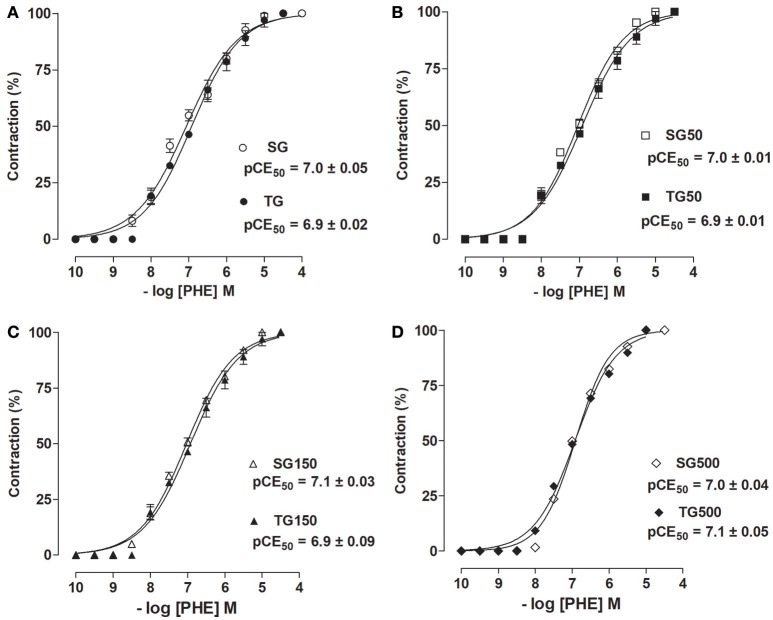
Contractile effect of PHE in SG (○), TG (•) **(A)**, SG50 (□), TG50 (■) **(B)**, SG150 (▵), TG150 (▴) **(C)**, SG500 (♢), and TG500 (♦) **(D)** groups in rat aorta in the absence of endothelium. The symbols and vertical bars represent the mean and SEM, respectively (*n* = 5).

The contractile response induced by PHE in rat aorta without functional endothelium was unchanged with strength training alone when compared to sedentary animals (pCE_50_ = 6.9 ± 0.01 vs. 7.0 ± 0.01, respectively) (Figure 4A). Additionally, there was also no change in the trained groups supplemented with *S. platensis* at doses of 50, 150, or 500 mg/kg when compared to sedentary groups supplemented with 50 (pCE_50_ = 7.0 ± 0.01 vs. 6.92 ± 0.01, respectively, Figure 4B), 150 (pCE_50_ = 6.9 ± 0.09 vs. 7.1 ± 0.03, respectively, Figure 4C) and 500 mg/kg (pCE_50_ = 6.9 ± 0.04 vs. 7.0 ± 0.04, respectively, Figure 4D). Similarly, the contractile efficacy of PHE was not altered by either strength training or supplementation with *S. platensis* in the absence of functional endothelium.

### Effect of strength training and supplementation with *S. platensis* on relaxation induced by ACh

**Figure 5:**
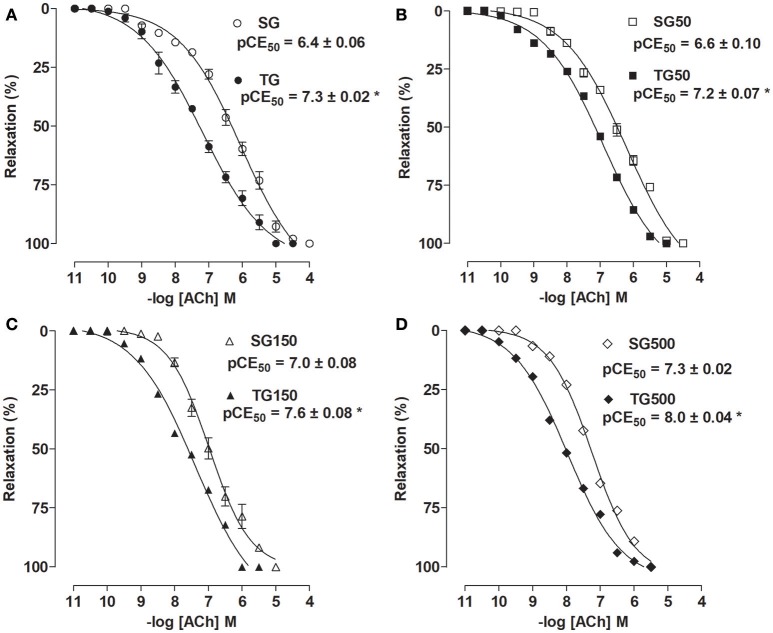
Relaxing effect of ACh in SG (○), TG (•) **(A)**, SG50 (□), TG50 (■) **(B)**, SG150 (▵), TG150 (▴) **(C)**, SG500 (♢) and TG500 (♦) **(D)** groups in rat aorta. The symbols and vertical bars represent the mean and SEM, respectively (*n* = 5). Student's *t* test, *p < 0.05 (SG vs. TG, SG50 vs. TG50, SG150 vs. TG150, SG500 vs. TG500).

The ACh relaxing potency was increased when the sedentary animals received supplementation with *S. platensis* at 150 (pCE_50_ = 7.0 ± 0.08) and 500 mg/kg (pCE_50_ = 7.3 ± 0.02) when compared to SG and SG50 (pCE_50_ = 6.4 ± 0.06 and 6.6 ± 0.10, respectively), no change was observed in E_max_. Among the rats subjected to strength training, doses of 150 and 500 mg/kg increased the ACh relaxing potency (pCE_50_ = 7.6 ± 0.08 and 8.0 ± 0.04, respectively) compared to TG (pCE_50_ = 7.3 ± 0.02) and TG50 (pCE_50_ = 7.2 ± 0.07) (Figure 5).

When comparing the trained and sedentary groups, it was shown that the pCE_50_ values of ACh were higher in rats of TG (pCE_50_ = 7.3 ± 0.02) than among SG (pCE_50_ = 6.4 ± 0.06) (Figure 5A). Strength training potentiated the increase in relaxing activity induced by supplementation with *S. platensis* with all doses tested. The pCE_50_ values of ACh increased from 6.6 ± 0.10 (SG50) to 7.2 ± 0.07 (TG50) (Figure 5B), 7.0 ± 0.08 (SG150) to 7.6 ± 0.08 (TG150) (Figure 5C) and 7.3 ± 0.02 (SG500) to 8.0 ± 0.04 (TG500) (Figure 5D).

Furthermore, supplementation with *S. platensis* at a dose of 150 mg/kg in the sedentary group did not cause greater relaxing response compared to TG (pCE_50_ = 7.0 ± 0.08 vs. 7.3 ± 0.02, respectively). However, in TG150 there was an increase in ACh relaxation potency compared to TG and SG150 (pD_2_ = 7.6 ± 0.08 vs. 7.3 ± 0.02 and 7.0 ± 0.08, respectively). Moreover, in SG500, there was an increase in relaxation potency similar to that in TG (pCE_50_ = 7.3 ± 0.02 vs. 7.3 ± 0.02, respectively). However, in TG500, the increase in relaxation potency was higher than TG and SG500 (pCE_50_ = 8.0 ± 0.07 vs. 7.3 ± 0.02 and 7.3 ± 0.02, respectively). Strength training did not change the maximum relaxing effect induced by ACh (Figure 5).

### Effect of strength training and supplementation with *S. platensis* on the cumulative contractions induced by PHE in the absence and presence of L-NAME

**Figure 6:**
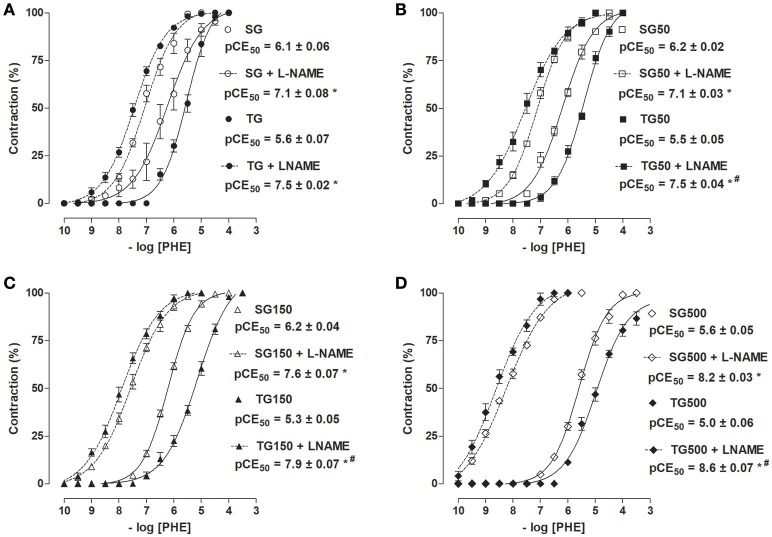
Contractile effect of PHE in SG (○), TG (•) **(A)**, SG50 (□), TG50 (■) **(B)**, SG150 (▵), TG150 (▴) **(C)**, SG500 (♢) and TG500 (♦) **(D)** groups in rat aorta in the absence and presence of L-NAME. The symbols and vertical bars represent the mean and SEM, respectively (*n* = 5). Two-way ANOVA followed by Bonferroni's post-test, *p < 0.05 (SG vs. SG + L-NAME, TG vs. TG + L-NAME, SG50 vs. SG50 + L-NAME, TG50 vs. TG50 + L-NAME, SG150 vs. SG150 + L-NAME, TG150 vs. TG150 + L-NAME, SG500vs. SG500 + L-NAME and TG500 vs. TG500 + L-NAME) and ^#^*p* < 0.05 (TG + L-NAME vs. SG + L-NAME, TG50 + L-NAME vs. SG50 + L-NAME, TG50 + L-NAME vs. SG50 + L-NAME and TG500 + L-NAME vs. SG500 + L-NAME).

The cumulative concentration-response curve to PHE in the presence of L-NAME was shifted to the left in the sedentary group and/or sedentary groups supplemented with 50, 150, and 500 mg/kg *S. platensis* when compared to SG in the absence of L-NAME (pCE_50_ = 7.1 ± 0.08, 7.1 ± 0.03, 7.6 ± 0.07, 8.2 ± 0.03, and 6.1 ± 0.06, respectively). Similar results were observed in rats subjected to strength training and/or supplementation with *S. platensis*, where there was an increase in contractile reactivity induced by PHE when compared to TG, TG50, TG150, and TG500 groups, in the presence of L-NAME, and TG in the absence of L-NAME (pCE_50_ = 7.5 ± 0.02, 7.5 ± 0.04, 7.9 ± 0.08, 8.6 ± 0.07, and 5.6 ± 0. 07, respectively) in aorta with functional endothelium. Similarly, sedentary animals treated with 150 and 500 mg/kg of *S. platensis* increased the contractile reactivity of aorta in the presence of L-NAME compared to TG50 and TG in the presence of this inhibitor (Figure 6).

When compared the sedentary groups with strength training groups, it was noted that the contractile potency was higher in TG + L-NAME compared to SG + L-NAME (pCE_50_ = 7.5 ± 0.02 vs. 7 1 ± 0.08, respectively) (Figure 6A). Similar results were observed for supplemented rats, both sedentary and trained. The pCE_50_ values of PHE increased from 7.5 ± 0.04 to 7.1 ± 0.03 (TG50 + L-NAME and SG50 + L-NAME, respectively) (Figure 6B), 7.9 ± 0.07 to 7.6 ± 0.07 (TG150 + L-NAME and SG150 + L-NAME, respectively) (Figure 6C) and 8.6 ± 0.07 to 8.2 ± 0.03 (TG500 + L-NAME and SG500 + L-NAME, respectively) (Figure 6D).

### Effect of strength training and supplementation with *S. platensis* on nitrite production in plasma and aortic rings

**Figure 7:**
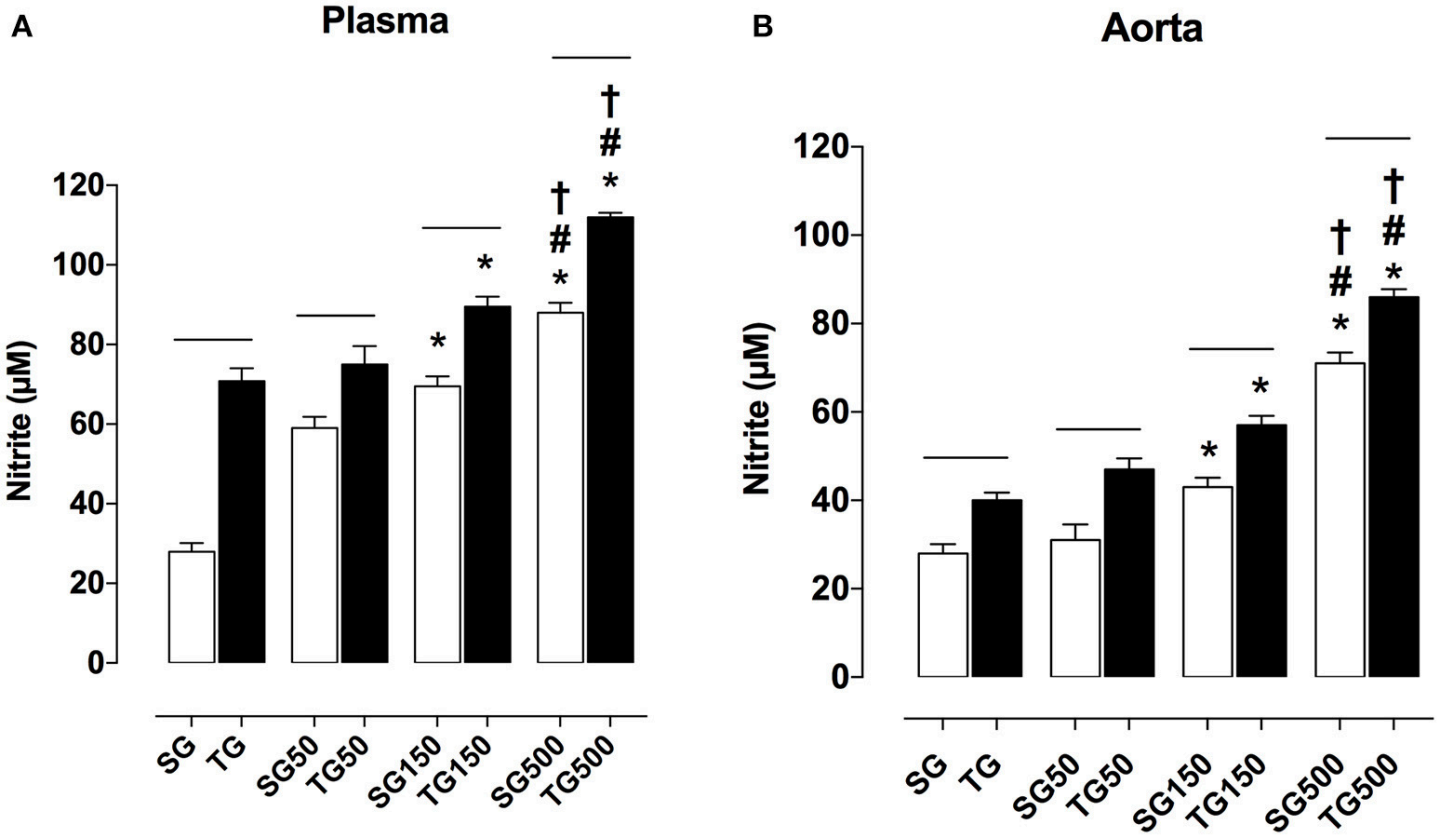
Concentration of nitrite in blood plasma **(A)** and aorta **(B)** from SG, TG, SG50, TG50, SG150, TG150, SG500, and TG500 groups, respectively. The columns and vertical bars represent the mean and SEM, respectively (*n* = 8). Two-way ANOVA followed by Bonferroni's post-test, *p < 0.05 (SG vs. SG150, SG vs. SG500, TG vs. TG150 and TG vs. SG500), ^#^*p* < 0.05 (SG50 vs. SG500 and TG50 vs. TG500), ^†^*p* < 0.05 (SG150 vs. SG500 and TG150 vs. TG500), ^−−^*p* < 0.05 (TG vs. SG, TG50 vs. SG50, TG150 vs. SG150 and TG500 vs. SG500).

Plasma nitrite concentration in all groups subjected to strength training was higher than in sedentary groups. The nitrite values in rats subjected only to strength training and sedentary rats were 70.0 ± 9.0 and 54.0 ± 11.0 μM, respectively. Strength training potentiated the increase in nitrite production with supplementation of *S. platensis* at all doses tested. The concentration of nitrite on plasma increased from 59.0 ± 8.0 (SG50) to 75.0 ± 13.0 μM (TG50), 70.0 ± 7.0 (SG150) to 89.0 ± 7.0 μM (TG150) and 88.0 ± 7.0 (SG500) to 112.0 ± 3.0 μM (TG500) (Figure 7A).

In the aorta, it was shown that in all groups subjected to strength training, the production of nitrite was higher than in sedentary groups. The nitrite concentration in rats subjected to strength training alone and sedentary rats was 40.0 ± 5.0 and 28.0 ± 6.0 μM, respectively. Strength training potentiated the increase in nitrite levels with supplementation of *S. platensis* with all doses tested. Aortic nitrite concentration values increased from 31.0 ± 10.0 (SG50) to 47.0 ± 7.0 μM (TG50), 43.0 ± 5.0 (SG150) to 57.0 ± 6.0 μM (TG150), and 71.0 ± 7.0 (SG500) to 86.0 ± 5.0 μM (TG500) (Figure 7B).

### Effect of strength training and supplementation with *S. platensis* on the production of malondialdehyde

**Figure 8:**
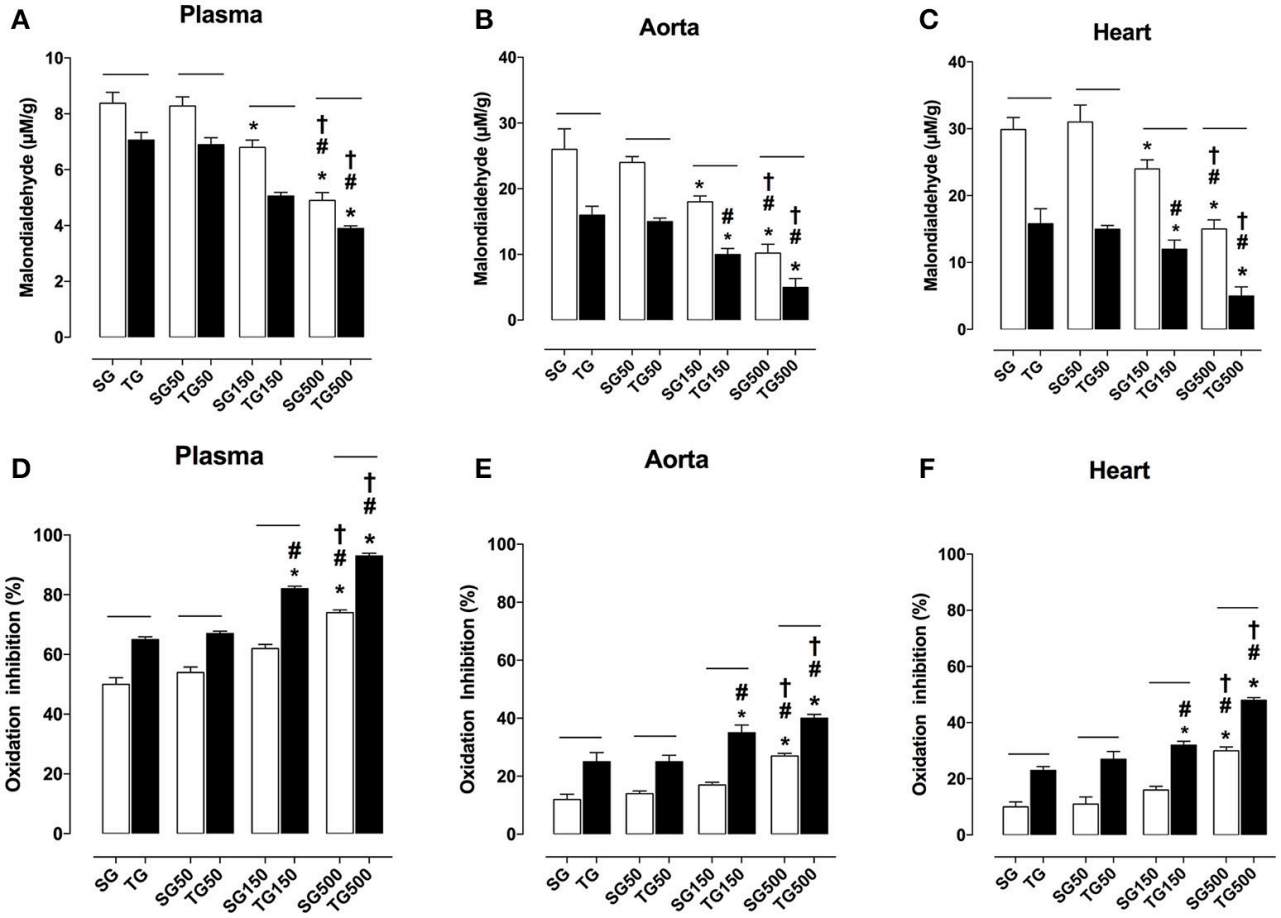
Lipid peroxidation in blood plasma **(A)**, aorta **(B)**, heart **(C)** and percentage of oxidation inhibition in blood plasma **(D)**, aorta **(E)**, heart **(F)** from SG, TG, SG50, TG50, SG150, TG150, SG500, and TG500 groups, respectively. The columns and vertical bars represent the mean and SEM, respectively (*n* = 8). Two-way ANOVA followed by Bonferroni's post-test, *p < 0.05 (SG vs. SG150, SG vs. SG500, TG vs. TG150 and TG vs. TG500), ^#^*p* < 0.05 (SG50 vs. SG150, SG50 vs. SG500, TG50 vs. TG150 and TG50 vs. TG500), ^†^*p* < 0.05 (SG150 vs. SG500 and TG150 vs. TG500), ^−−^*p* < 0.05 (TG vs. SG, TG50 vs. SG50, TG150 vs. SG150 and TG500 vs. SG500).

The MDA plasmatic concentration in all groups subjected to strength training was lower than in sedentary groups. The MDA values in rats subjected to strength training alone and sedentary rats were 7.1 ± 1.0 and 8.3 ± 1.1 nmol/L, respectively. Strength training potentiated the reduction in MDA levels with supplementation of *S. platensis* at all doses tested. MDA values decreased from 8.2 ± 2.0 (SG50) to 6.9 ± 2.0 nmol/L (TG50), 6.8 ± 0.7 (SG150) to 5.6 ± 0.4 nmol/L (TG150) and 5.0 ± 0.1 (SG500) to 3.9 ± 0.4 nmol/L (TG500) (Figure 8A).

Comparing the sedentary and exercised groups, strength training potentiated the reduction in MDA production in aorta with supplementation of *S. platensis* at all doses tested. MDA decreased from 26.0 ± 6.0 (SG) to 16.0 ± 3.0 μM/g (TG), 24.0 ± 2.0 (SG50) to 15.0 ± 2.0 μM/g (TG50), 18.0 ± 2.0 (SG150) to 10.0 ± 21.0 μM/g (TG150) and 10.0 ± 3.0 (SG500) to 5.0 ± 3.0 μM/g (TG500) (Figure 8B).

Similarly, in the heart, it was shown that training potentiated the decrease in MDA when TG was compared to SG (16.0 ± 6.0 vs. 29.0 ± 4.0 μM/g), TG50 was compared to SG50 (15.0 ± 2.0 vs. 31.0 ± 6.0 μM/g), TG150 was compared to SG150 (12.0 ± 3.0 vs. 24.0 ± 3.0 μM/g) and TG500 was compared to SG500 (5.0 ± 3.0 vs. 15.0 ± 3.0 μM/g) (Figure 8C).

### Effect of strength training and supplementation with *S. platensis* on antioxidant activity

In all groups subjected to strength training, the plasma antioxidant activity was higher than in sedentary groups. The percentage values in rats subjected to strength training alone and sedentary rats were 65.0 ± 2.0 and 50.0 ± 5.0%, respectively. Strength training enhanced the percentages of antioxidant activity with the supplementation of *S. platensis* at all doses tested. Values increased from 54.0 ± 4.0 (SG50) to 67.0 ± 2.0% (GT50), 62.0 ± 3.0 (SG150) to 82.0 ± 2.0% (GT150), and 74.0 ± 2.0 (SG500) to 93.0 ± 2.0% (GT500) (Figure 8D).

In aorta, comparing the sedentary and exercised groups, strength training enhanced antioxidant activity with the supplementation of *S. platensis* at all doses tested. Values increased from 12.0 ± 4.0 (SG) to 25.0 ± 7.0% (GT), 14.0 ± 2.0 (SG50) to 25.0 ± 5.0% (GT50), 17.0 ± 2.0 (SG150) to 35.0 ± 6.0% (GT150), and 27.0 ± 2.0 (SG500) to 43.0 ± 3.0% (GT500) (Figure 8E).

Similarly, in the heart, it was shown that training potentiated the antioxidant activity when TG was compared to SG (23.0 ± 3.0 vs. 10.0 ± 4.0%), TG50 was compared to SG50 (27.0 ± 6.0 vs. 11.0 ± 6.0%), TG150 was compared to SG150 (32.0 ± 3.0 vs. 16.0 ± 3.0%) and TG500 was compared to SG500 (48.0 ± 2.0 vs. 30.0 ± 3.0%) (Figure 8F).

## Discussion

This study showed that chronic supplementation with *Spirulina platensis* at doses of 150 and 500 mg/kg decreased contractile reactivity to PHE and increased relaxing activity to ACh, but when these doses were used in association with strength training, there was potentiation of the reduction in contractile reactivity to PHE and increase in relaxing activity to ACh. Also, it was shown that the factors that accompanied this improvement in reactivity involve the release of nitric oxide and reduction in oxidative stress and systemic inflammation. Accordingly, these data demonstrated for the first time that the synergistic action between strength training and *S. platensis* results in improving vascular reactivity.

First, to confirm the exercise protocol effectiveness, it was determined that there was a decrease in the time that the animal used to perform the jumps in each series of exercise in the last week of the protocol when compared to the first session, demonstrating the efficacy of the training protocol (Figure 1). Furthermore, there was no significant increase in LDH or CK activity in any of the trained groups when compared to control group (Figure 2), indicating that despite that strength training was in steps of progressive loads, it did not elicit an exaggerated stress in skeletal muscle, which could lead to the phenomenon called overtraining (Smith, 2004).

The improvement in vascular reactivity, represented by the reduction in the contractile activity (Delbin et al., 2012; Okudan et al., 2012) and increased relaxing activity (Valgas Da Silva et al., 2014) is already well established for the aerobic exercise modality. Concerning the role of strength training in vascular reactivity, three studies have demonstrated improvement in relaxing activity (Figard et al., 2006; Harris et al., 2010; Araujo et al., 2013) and only in one of them the contractile reactivity with a reduction in this activity was measured (Araujo et al., 2013).

Despite the changes in vascular responsiveness as a result of the resistance exercise investigated, training protocols are quite different and far from what could be extrapolated to humans. Figard et al. (2006) performed an isometric strength training for a period of 14 weeks in rats and evaluated the relaxing activity in aortic rings through cumulative curves to ACh. Harris et al. (2010) subjected aged rats to a strength training that consisted in climbing a ladder with a workload tied to the base of their tail for 6 weeks and the femoral artery relaxing activity was evaluated by concentration-response curves to ACh. More recently, Araujo et al. (2013) subjected hypertensive animals to a four-week training, held three times a week with a continuous load of 50% of 1 maximum repetition and 3 sets of 10 repetitions prompted by electrical stimulation through electrodes attached to tail, and after this training, the vascular reactivity to PHE was measured in the mesenteric artery. Although they were different methodological models with relaxing activity being significantly improved, the results are still very scarce in the study and none have been done more than one test.

Meanwhile, in this study, as well as simultaneously evaluating the relaxing and contractile activity, the training protocol used was similar to that practiced by humans in real conditions of physical activity, with jumping exercises, simulating the squat exercise (Garber et al., 2011).

This protocol has already been proposed to induce muscle hypertrophy in rats (Marqueti et al., 2006). Thus, these data showed that the improved relaxing effect and reduced contractile response already established in the literature for aerobic training was also observed for the strength training adopted in this study with characteristics closer to that adopted by humans. Thus, our results suggest that a better vasodilation and inhibition of vasoconstriction (Figures 3–5) could explain the reduction in blood pressure that has been observed in humans who undergo strength training programs, as well described by others (Brito et al., 2011, 2014) and in published meta-analyses (Anunciação and Polito, 2011). However, to confirm this assumption, this method should be used again in future studies with hypertensive rats.

Considering that (Huang et al., 2005) demonstrated that oral administration of polysaccharides of *S. platensis* at doses of 12.261, 36.783, and 110.349 mg/kg, during six-week, in diabetic rats improved vascular reactivity. Indeed, the greatest reductions in contractile potency (Figure 3) and the largest increases in relaxing potency were identified (Figure 5) in the trained groups supplemented concurrently with doses of 150 and 500 mg/kg of *S. platensis*, indicating a synergistic effect between training and nutritional supplementation with *S. platensis*. Additionally, the data obtained in this study demonstrated that supplementation at doses of 150 and 500 mg/kg was able to improve the reduction in contractile response and increase in relaxation that had been demonstrated with the protocol consisting only of strength training.

The constituents present in *S. platensis*, such as phycocyanin, can chronically increase the expression of endothelial NOS and consequently promote a greater bioavailability of NO (Ichimura et al., 2013). In previous studies consisted of strength training protocols, two confirmed that the improvement in relaxing effect occurred by mechanisms dependent on endothelium via increased production of NO, a relaxant factor derived from endothelium (Figard et al., 2006) and the increased of Hsp90 expression, a regulator of eNOS activity and binding (Harris et al., 2010). Although Araujo et al. (2013) found for the first time a reduction in contractile reactivity in response to strength training, the mechanism involved was not investigated, but it was noted that the decrease in absence of elevated blood pressure under the conditions of this study, appeared to involve a regulatory vasoconstrictor mechanism.

In this study, it was sought to determine whether the reduction in contractile activity would also be dependent on mechanisms mediated by the endothelium. Accordingly, it was not observed a decrease in the contractile response in rats subjected to the training and supplementation with *S. platensis* in the absence of the endothelium (Figure 4), demonstrating that the contractile activity inhibition promoted both by strength training and *S. platensis* was dependent on endothelium.

In the presence of L-NAME, a non-selective competitive inhibitor of NOS, it was observed an increase in PHE contractile response elicited by training and supplementation with *S. platensis* (Figure 6). It is established that the practice of exercise training and bioactive compounds present in natural products stimulate the increase in NO bioavailability (Spier et al., 2004; Durrant et al., 2009; Corbi et al., 2016), and consequently, improve endothelium-dependent vasodilation (DeVan et al., 2013; Luttrell et al., 2013). Based on the results obtained by this study and the literature, the participation of the NO pathway on the beneficial effects promoted by exercise training and food supplementation with *S. platensis* can be suggested.

The endothelium role in the reduction of contractile activity was accompanied by an increase in the production of nitrite in plasma and aorta at doses of 150 and 500 mg/kg of *S. platensis* and in trained rats. These effects were potentiated when training and food supplementation with *S. platensis* were used concomitantly (Figure 7). Briefly, nitrite concentrations for SG150 and SG500, TG150, and TG500 in plasma and aorta were significantly higher; indicating that food supplementation with the powder of *S. platensis* increased the bioavailability of NO in plasma and tissue, and enhanced the effect of strength training (Figure 7). Similarly, the study pioneered in directly investigating the vasocontractile function improvement in response to strength training and its relationship with the production of NO, which was enhanced by food supplementation with *S. platensis*.

Previously, it was shown that endothelial dysfunction is mainly a result of impaired NO availability leading to increased production of ROS (Tang and Vanhoutte, 2010). Accordingly, the MDA production was determined, a lipid peroxidation marker, as well as the oxidation inhibition percentage, an antioxidant activity marker, in samples of the aorta and cardiac muscle. With regard to lipid peroxidation, it was shown a reduction in MDA potentiated when animals were subjected to training and food supplementation with *S. platensis* at doses of 150 and 500 mg/kg (Figures 8A–C). In relation to antioxidant activity, it was demonstrated that the oxidation inhibition percentage was increased with training and supplementation with *S. platensis* (Figures 8D–F). Thus, these results were associated with a decrease in oxidative stress observed in response to strength training and supplementation with *S. platensis*.

The phycocyanin present in *S. platensis* stands out for its high antioxidant capacity and potent scavenging of free radicals due to its stability (Bhat and Madyastha, 2000) and inhibitory effect on the formation of superoxide radicals by reducing the expression of subunit p22^phox^ of nicotinamide adenine dinucleotide phosphate of oxidase (McCarty, 2007; Kuddus et al., 2013). Moreover, the carotenoids are also essential for the regulation of SOD and catalase (CAT), and blocking free radicals by chelation of metal ions, preventing lipid peroxidation (Cuvelier, 2001). Furthermore, B vitamins and vitamin E also act as antioxidants via capture of radicals and metal chelating agents (Cuvelier, 2001).

According to this study results, it is worth investigating the potential of *S. platensis* as a nutraceutical and food supplement, to bring an alternative to the region's economy. However, for this to happen, further studies are needed to add to these presented data, for example, in animal models of certain diseases such as hypertension, diabetes and/or obesity.

Pharmacological studies using an experimental model smooth muscle have sought to identify effective therapeutic strategies in the treatment of various diseases such as cardiovascular diseases. Therefore, this study is the first to demonstrate that the use of *Spirulina platensis* in association with resistance exercise practice is able to improve the smooth muscle contractile response, to promote increased antioxidant activity and decreased pro-oxidant activity, indicating that the association between these therapies can be a non-pharmacological instrument for the treatment and prevention of diseases arising from the smooth muscle.

Therefore, it was concluded that strength training in association with food supplementation of *S. platensis* improves muscle performance and protects against muscle damage, oxidative stress, and inflammation. In addition, it decreased the contractile reactivity induced by PHE and increased the relaxing response to ACh in the aorta with endothelium. These responses were mediated by factors dependent on vascular endothelium, such as increased NO bioavailability in plasma and aorta, reduced lipid peroxidation and increased antioxidant activity in plasma, aorta, and heart.

## Author contributions

AB, AS and BdS developed the hypothesis and experimental design. AB, IdS, and LA analyzed the data and wrote the manuscript. AB, IdS, and PF performed the experimental work. RT, AdS, GF, RP, MM, and LA contributed to the *in vivo* work. RS and MS contributed to the *in vitro* work.

### Conflict of interest statement

The authors declare that the research was conducted in the absence of any commercial or financial relationships that could be construed as a potential conflict of interest.
